# Neurosyphilis presenting with dementia, chronic chorioretinitis and adverse reactions to treatment: a case report

**DOI:** 10.4076/1757-1626-2-8334

**Published:** 2009-09-01

**Authors:** Shima Mehrabian, Margarita Radoslavova Raycheva, Elena Petrova Petrova, Nikolay Konstantinov Tsankov, Latchezar Dintchov Traykov

**Affiliations:** 1 Department of Neurology1, Georgi Sofiiski str, 1431 SofiaBulgaria; 2 Department of Dermatology and Venereology1, Georgi Sofiiski str, 1431 SofiaBulgaria

## Abstract

Neurosyphilis results from infection of the brain, meninges or spinal cord by Treponema pallidum and develops in about 25%-40% of persons who are not treated for syphilis. This article reports a rare case of active neurosyphilis with mild dementia, chronic chorioretinitis, and hearing loss. During the treatment with Penicillin, a rare combination of complications such as Jarisch-Herxheimer and Hoigné reactions were observed.

The clinical feature is characterized by a slow progressive cognitive decline and behavior changes for the last 2 years. Neuropsychological examination revealed mild dementia (MMSE = 23) with impaired memory and attention and executive function. Left sided chronic chorioretinitis and hearing loss were documented. High dose intravenous penicillin therapy was complicated by Jarisch-Herxheimer and Hoigne reactions. During the follow up examinations at 6 and 12 months, the clinical signs, neuropsychological examination, and cerebrospinal fluid (CFS) samples showed improvement of dementia, CSF findings, and hydrocephalus.

In conclusion, this atypical presentation of neurosyphilis in combination with rare complications of treatment is worthy of attention. Neurosyphilis should be part of the differential diagnosis of each patient showing cognitive deterioration and behaviour disturbances.

## Introduction

Neurosyphilis is recognized as playing an important role in the evolution of modern neurology, for about 100 years. It results from infection of the brain, meninges or spinal cord by Treponema Pallidum and develops in about 25%-40% of persons who are not treated for syphilis. The clinical presentations of neurosyphilis are extremely varied and, for practical purposes, can be divided into early and late neurosyphilis. Clinical manifestations of early neurosyphilis include meningovascular diseases, acute and subacute myelopathy, brainstem or cranial nerve abnormalities, and vestibular and ocular disease. Although overlap could be substantial, late neurosyphilis tends to affect the brain and spinal cord parenchyma, typically presenting as dementia, tabes dorsalis, general paresis, sensory ataxia or bowel/bladder dysfunction [1].

Cognitive decline is one of the manifestations of late syphilis. However, mild cognitive impairment could be observed in early stages of neurosyphilis [2]. It should be noted that before his most celebrated discovery, Alois Alzheimer had dedicated several years of his life to the study of the neuropathology of syphilis, which was the theme of his post-doctoral thesis (Habilitationsschrift) [3].

This article reports a case of neurosyphilis with mild dementia, chorioretinitis and hearing loss. Clinical, neuropsychological and neuro-imaging follow up together with examination of cerebrospinal fluid (CSF) were performed at the sixth and twelfth month.

## Case presentation

A 56-year-old man (Bulgarian patient of Caucasian origin) sought memory consultation at the University Hospital Alexandrovska in Sofia because of reduced concentration and attention, memory loss, and behavior changes such as apathy, anxiety, and agitation within the last 2 years. Additionally, the patient reported occasional dizziness, ataxia, and hearing loss with tinitus. There was no history of skin lesions or symptom of Argyll-Robertson. Impaired auditory acuity was found, more pronounced in the left ear. During the motor coordination examination the patient performed the finger to nose test with dysmetria in the left hand. Evidence of instability was observed during the examination of joint position sense. The patient reported bilateral paresthesia in the distal parts of the extremities. No pathologic reflexes were found.

Laboratory workups including a complete blood count and differential, serum electrolytes and glucose, liver and renal function tests, thyroid function tests, serum B12 and folate levels, and an ECG were normal. The serum and CSF test for human immunodeficiency virus (HIV) was negative. The diagnosis of active neurosyphilis was based on positive results of Venereal Disease Research Laboratory test - Treponema pallidum. Hemagglutination assay (VDRL-TPHA) reactions in blood and CSF samples. In addition, CSF analysis showed pleocytosis, elevated protein levels, and positive oligoclonal band. Cerebrospinal fluid protein concentration was 80 mg/dl, CSF-leukocyte count was 39 cells/mm^3^ (82% mononuclear cells), serum-VDRL 1:128, and serum-TPHA 1:2560. Examinations revealed vestibulopathy, hearing loss and chronic chorioretinitis on the left without vision loss or eye pain. Cognitive status was evaluated by the mini-mental state examination (MMSE) and a detailed neuropsychological battery for assessment of memory, language and executive functions, attention, and concentration. The examination documented mild dementia (MMSE = 23) with moderate amnestic and dysexecutive syndrome. Computed tomography (CT)/magnetic resonance imaging (MRI) of brain demonstrated moderate ventricular dilatation and mild cerebral atrophy. The patient received a course of intravenous Penicillin G 4 x 2000000 UI/daily for 20 days.

After the first use of Penicillin, Jarish-Herxheimer Reaction (JHR) was observed along with a high temperature (38.6°C) for several hours. That was treated with antipyretics. After the second use of Penicillin, Hoigné reaction developed; the patient became pale with agitation, anxiety, fear of death, palpitation, and vision and hearing hallucinations.

During the follow up examinations at 6 and 12 months, the clinical signs, neuropsychological, and neuroimaging findings showed improvement. The MMSE done 6 and 12 months after the treatment scored 26 and 27, respectively (Table 1). Improvement was also noted in the activities of daily living assessment, while behavioural disturbances were disappeared. Six months later, the patient’s CSF protein level was 53 mg/dL, with 9 mononuclear cells/mm^3^, and VDRL testing of CSF yielded positive results. Antisyphilitic therapy brought about mild improvement in the CT/MRI abnormalities.

## Discussion

A case of active neurosyphilis has been presented with dementia, behavior changes, chronic chorioretinitis, and hearing loss in combination with rare complications of treatment.

After the appearance of acquired immunodeficiency syndrome (AIDS) in 1981, the occurrence of neurolues in HIV infection is the reason for the increased number of new cases in developed countries [1]. Neurosyphilis is a disease still occuring nowadays, and because of its clinical polymorphism, must be considered as a differential diagnosis in a number of neurological and psychiatric illnesses [4].

Detailed neuropsychological examination revealed that because of syphilitic vasculitis with lacunar infarctions, patients with neurosyphilis have earlier and more severe executive dysfunction in comparison to AD patients. Dementia responds to treatment with Penicillin as a manifestation of neurolues and is considered a “potentially reversible” dementia [5]. Abnormalities in neurosyphilis detected through MRI include generalized atrophy, focal lesions, nonspecific white matter changes, and extra-axial enhancement indicating meningitis [6]. The clinical picture of meningovascular syphilis may be associated with focal neurological signs of cerebral arteritis. Inflammation of the leptomeninges may impede circulation of the CSF at different levels, giving rise to noncommunicating or unusually, to communicating hydrocephalus [7]. This patient had moderate hydrocephalus at the time of diagnosis. There was a mild improvement in hydrocephalus after treatment documented by brain CT at twelfth month.

Left-sided chronic chorioretinitis without pain or discomfort was revealed in this patient. Ophtalmological investigation did not reveal improvement after treatment with Penicillin. Chronic diffuse chorioretinitis is a feature of the clinical spectrum of posterior syphilitic uveitis. The frequent association of syphilitic posterior uveitis with neurosyphilis and the analogous spirochetal sequestration beyond the blood-brain and the blood-ocular barriers suggest that all patients with syphilitic posterior uveitis, irrespective of ocular disease intensity, should undergo CSF evaluation and be treated with Penicillin regimens appropriate for neurosyphilis [8].

Syphilitic otitis would appear as asymmetric deafness and tinnitus. Syphilis can be found as the cause in about 7% of patients with otherwise unexplained hearing loss [6], and is the likely cause of hearing loss and tinnitus in this patient. After treatment with Penicillin, the patient reported improvement in tinnitus.

High dose intravenous penicillin therapy was complicated by JHR and Hoigne reactions. To the authors’ knowledge, the combination of these reactions in Penicillin therapy of neurosyphilis is not reported in literature. Penicillin was first introduced for the treatment of syphilis in 1943, and has maintained the only recommended antibacterial agent for neurosyphilis [9]. The JHR is a self-limited febrile inflammatory response to treatment of a number of bacterial infections, and louse-born relapsing fever. Within the context of syphilis therapy, the JHR begins typically 1 ± 2 h after the commencement of penicillin and is characterised by fever, chills, myalgias, headache, hyperventilation, haemodynamic instability, and exacerbation of skin lesions [6]. In 1959, R. Hoigne described the first cases of pseudo-anaphylactic reactions induced by intramuscular administration of procaine penicillin G [10]. Hoigne’s syndrome is currently considered a pseudoanaphylactic or pseudoallergic reaction following intramuscular and aqueous procaine penicillin administration. This disorder is characterized predominantly by neuropsychiatric alterations including severe psychomotor agitation, as well as alterations of consciousness and seizures [11]. It must be differentiated from authentic anaphylactic shock as a result of penicillin. The distinction is important from a therapeutic viewpoint because Hoigné’s syndrome allows continuation of treatment, whereas it is absolutely contraindicated in anaphylactic shock.

## Conclusion

This atypical presentation of neurosyphilis in combination with rare complications of treatment is noteworthy. The clinical picture of meningeal and meningovascular forms and atypical feature without skin lesions delay the diagnosis and obligate the laboratory confirmation of neurosyphilis. Neurosyphilis should be part of the differential diagnosis of each patient showing deterioration in cognition and behaviour disturbances. During follow-up, neuropsychological assessments and CSF examinations are useful instruments to measure cognitive decline and response to treatment.

## Abbreviations

AIDS: Acquired Immunodeficiency Syndrome
CSF: cerebrospinal fluid
CT: computed tomography
HIV: Human immunodeficiency virus
JHR: Jarish-Herxheimer Reaction
MMSE: Mini Mental State Examination
MRI: Magnetic Resonance Imaging
VDRL-TPHA: Venereal Disease Research Laboratory test - Treponema Pallidum. Hemagglutination Assay
